# Compliance with COVID-19 preventive measures is high among university-level students in Québec, Canada

**DOI:** 10.14745/ccdr.v48i78a07

**Published:** 2022-07-07

**Authors:** Yohann Pilon, Radu Turcitu, Robert Allard

**Affiliations:** 1 School of Medicine, Faculty of Medicine and Health Sciences, McGill University, Montréal, QC; 2 Département de microbiologie, infectiologie et immunologie, Faculté de Médecine, Université Laval, Québec, QC; 3 Faculté de Médecine, Université de Montréal, Montréal, QC; 4 Department of Epidemiology, Biostatistics and Occupational Health, Faculty of Medicine and Health Sciences, McGill University, Montréal, QC

**Keywords:** coronavirus disease, COVID-19 surveillance, COVID-19 preventive measures, compliance

## Abstract

**Background:**

Canada’s nationwide lockdown to curb coronavirus disease 2019 (COVID-19) infections affected many sectors of activity, including universities. During the 2020–2021 academic year, all students were forced to follow their lectures from home and the only in-person activity permitted to Québec university level students was to study in designated spaces of campus libraries where COVID-19 preventive measures were in place and mandatory at all times for all staff and students. The objective of this study is to evaluate university-level students’ compliance with COVID-19 preventive measures in a Québec campus library.

**Methods:**

A direct in-person evaluation by a trained observer was put in place to assess students’ compliance with COVID-19 preventive measures defined as proper mask wearing and 2 meter distancing. Measurements were made each Wednesday, Saturday, and Sunday at 10 a.m., 2 p.m., and 6 p.m. from March 28 to April 25, 2021, in a university library in Québec, Canada.

**Results:**

Students’ compliance with COVID-19 preventive measures was high overall (78.4%) and increased over the weeks, with differences between weeks, weekdays, and time of day. Non-compliance was lower on weeks three and four of the assessment compared with week one, and higher on Sunday compared with Wednesday. Differences seen throughout the day were not statistically significant. Non-compliance with physical distancing was rarely seen.

**Conclusion:**

Most university-level students are compliant with COVID-19 preventive measures in a Québec university library: an encouraging behaviour from a public health perspective. These findings may support public health authorities or university administrators in decisions regarding different COVID-19 preventive measures directed to different universities settings, as this method can be applied to focused, rapid observational studies and can lead to data of sufficient statistical power.

## Introduction

On March 11, 2020, the World Health Organization declared the coronavirus disease 2019 (COVID-19) a pandemic ((1)). COVID-19 is transmitted predominantly by inhalation of fine aerosols, particles and droplets, direct exposition of mucous membranes of the mouth or eye to respiratory droplets and to a lesser extent through contact with contaminated surfaces ((2)). It has been established that the risk of transmission can be reduced using well-fitting masks and by physical distancing, surface disinfection, adequate ventilation, and avoidance of crowded spaces ((2–6)).

Studying in designated spaces, such as libraries, remained one of the very few in-person activity accessible to students in Québec, Canada who were enrolled in university during the 2020–2021 academic year ((7,8)). During the 2021 winter semester, designated study spaces in universities in this province were adapted for student safety by mandating both proper surgical mask use and physical distancing at all times ((9–17)). In-person activities for university-level students have been proven to be important for education, professional networking and socialization, and for good physical and mental health (18). Thus, knowledge of students’ compliance rate with COVID-19 preventive measures (COPM) in universities will help university administrators better apply public health policies for a rapid and safe return to in-person activities.

One way of evaluating the efficacy of a measure put in place is to assess compliance with it. Direct observation by trained observers has been used when evaluating the hand hygiene compliance of healthcare workers and remains the gold standard method for monitoring compliance towards similar measures (19). Many research teams have demonstrated the ability to evaluate compliance with COPM by using a direct in-person observation method (20–23); however, studies evaluating compliance with all COPM in university establishments are currently lacking.

In this study, we evaluated the compliance rate of students with COPM implemented in designated study spaces of a university. The hypothesis was that compliance by the students would be high. Indeed, previous studies of Canadians over 18 years of age have shown high compliance rate with COPM and high level of knowledge about mask use among university level students in Canada (24,25).

## Methods

### Library setting

The study was conducted in a university library that extends over seven stories, with a total capacity of 852 (pre-pandemic) and 313 (during pandemic) individual study workstations. These numbers exclude team study rooms and classrooms within the library. This library offers various services for students and holds collections in literature and human sciences. Library employees were assigned to remind each student of the COPM at the entrance of the library while also providing a procedural mask and alcohol hand disinfectant. An online reservation system was put in place to monitor arrival of the students and allocate them to a study space. Moreover, security agents regularly patrolled the entire library to ensure compliance with the COPM. Students were asked to properly wear the procedural masks (covering both mouth and nose) at their designated space at all times, even when studying alone.

### Observations

Observations were conducted using the in-person trained observer method. This method was favoured over a self-reported survey method because the risk of information bias is lower. We used a sampling methodology based on published studies and on a report released by Resolve to Save Lives (20,23,26,27)). The two observers were themselves students. They self-trained to 1) evaluate proper mask wearing and 2) estimate a 2 meter distance to reduce the risk of variability between observers. Observers were compliant with the COPM put in place by the library (properly wearing a mask at all times and physically distancing).

Observations were restricted to students who were sitting or were near the area of their designated study space (i.e. at desks in open spaces of the library). Gender and age were not considered, as the goal of the study was to evaluate university level students’ compliance with the COPM, which applied to students from any gender and all ages. Each observer was assigned a floor in the library and circulated on that floor directly observing each student. All observers participated in every time point to eliminate any interobserver confounding of week, weekday or time of day effects. Proper mask wearing and physical distancing were evaluated for each student observed and were assessed as being met or not. Students meeting both criteria were counted as complying with the COPM while students meeting one or no criterion were counted as non-complying with the COPM. Students circulating in the library were not included among the observations because 1) they did not represent a significant number of observations and 2) it would increase the complexity of the task, therefore increasing the risk of counting error for the observers. Students in individual study rooms (enclosed rooms with a door) were also excluded since the use of face masks was not required there. Moreover, since it is known that the Hawthorne effect can falsely increase compliance (28,29), observers counted students’ behaviour change, such as properly placing the mask or physical distancing, in the presence of the observer as non-compliance with the COPM. Data collection was done by filling out standardized paper forms that were then entered electronically into an Excel (version 2104; Microsoft Office Professional Plus 2019) spreadsheet after each period of observation. Observations were carried out at three time points (10 a.m., 2 p.m. and 6 p.m.) every Wednesday, Saturday, and Sunday over a period of four weeks from March 28 to April 25, 2021. Frequency of observations was spread evenly during the day and weekday to control for possible behaviour and occupancy variability. We chose to favour full coverage of weekends over weekdays for observations as it was assumed that lectures were given during the week, which would result in fewer students studying in the library. This 1-month time frame was chosen for observations as it covered most undergraduates’ end of semester exams and study period; a period when students tend to study more.

### Statistical analysis

Binary univariate and multivariate logistic regression analyses were performed. Multivariate regression included all three independent variables. Odds ratios and their confidence interval (CI) were calculated as un-adjusted (OR) and adjusted (AOR) for univariate and multivariate analysis respectively. SPSS version 27.0 software (SPSS, Inc., Chicago, Illinois, United States) was used for all statistical analyses.

### Ethics

Exemption from ethical review was granted by the Institutional Review Board of the university where observations were made. Disclosure of the university where the study took place was not permitted by the Institutional Review Board.

## Results

A total of 2,109 students were observed over 39 observation time points (13 days of observation, 3 observation time points per day), 27 during the weekends (on Saturdays and Sundays) and 12 during the week (on Wednesdays). All observations are summarized in **Table 1** and **Annex** (**Table S1** and **Table S2**).

**Table 1:** Non-compliance with COVID-19 preventive measures^a^ by week, weekday and time of day, Canada, Québec, March–April 2021

Characteristics	Number (%) of students							
	Non-compliance (N=456, 21.6%)		Compliance (N=1,653, 78.4%)		Non-compliance OR		Non-compliance AOR	
	n	%	n	%	OR	95% CI	AOR	95% CI
Week								
1	113	28.2	288	71.8	1	N/A	1	N/A
2	110	27.4	292	72.6	0.96	0.71–1.31	0.99	0.72–1.35
3	89	16.2	461	83.8	0.49	0.36–0.67	0.51	0.37–0.70
4	144	19.0	612	81.0	0.60	0.45–0.80	0.56	0.42–0.75
Weekday								
Wednesday	118	18.8	509	81.2	1	N/A	1	N/A
Saturday	133	20.9	533	79.1	1.08	0.82–1.42	1.09	0.82–1.43
Sunday	205	24.4	611	75.6	1.45	1.12–1.87	1.53	1.18–1.99
Time of day								
10 a.m.	84	21.8	302	78.2	1	N/A	1	N/A
2 p.m.	203	18.7	883	81.3	0.83	0.62–1.10	0.81	0.61–1.08
6 p.m.	169	26.5	468	73.5	1.30	0.96–1.75	1.22	0.90–1.65

Abbreviations: AOR, adjusted odds ratio; COVID-19, coronavirus disease 2019; N/A, not applicable; OR, un-adjusted odds ratio

^a^ COVID-19 preventive measures refers to both proper masking and adequate physical distancing

### Non-compliance by week, weekday and time of day

Observations were first grouped as compliant or not with the COPM: 1,653 (78.4%) students were compliant whereas 456 (21.6%) students were non-compliant (Table 1).

Binary logistic regressions showed that student’s non-compliance with the COPM was dependent on the week, weekday, and time of the day. Non-compliance with COPM was lower on weeks three and four when compared with week one (AOR=0.51 and AOR=0.56, respectively) and non-compliance with COPM was higher on Sunday compared with Wednesday (AOR=1.53). As for time of day, non-compliance with COPM was lower at 2 p.m. (AOR=0.81) and higher at 6 p.m. (AOR=1.22) when compared with 10 a.m., but these differences were not statistically significant.

Similar results were seen for non-compliance with proper mask use alone (Table S1). Physical distancing was rarely inadequate; n=50 (2.4%) and n=14 (0.6%) were observed not physically distanced whether properly or improperly wearing the mask, respectively (Table S2).

## Discussion

The observed 78.4% level of compliance with COPM is close to the 80% threshold suggested as necessary to reduce the spread of COVID-19 (30). Compliance with COPM increased over the weeks, probably because higher numbers of students in the library lead to increased enforcement of COPM. Observers noted that enforcement of COPM was variable and might not have been consistent over time. Indeed, our study has been conducted at the end of the 2021 winter semester: a time when students tend to study more because end-term exams are approaching. Students were less likely to be compliant with the COPM on Sunday.

Our study was carried out one month prior to the widespread vaccination availability in Québec, Canada (which began April 30, 2021). This timing could have positively affected students’ attitude towards COPM (31,32). A direct observation method has proven to be valuable in an acute period of the pandemic as it allowed for rapid evaluation of compliance in a large sample. This is especially valuable when public health measures are changing rapidly, as this method can be quickly implemented as well. It also eliminates reporting bias, a limitation that is often encountered in survey-based studies (33). This method proved to be challenging as it does not allow much flexibility in the observers’ work schedule. Indeed, the distribution of observation time points across the day (10 a.m., 2 p.m., and 6 p.m.) required the observers to be disciplined in their work schedules and for them to determine the circulating library floors before each observation time point, as observations must take place at each established time point to prevent compromising the robustness of the study. Future studies could evaluate masking behaviour across multiple universities using the same method. Indeed, now that vaccination coverage is higher, and that the current pandemic situation has changed significantly (34), compliance with masking and physical distancing mandates could differ from those we observed, suggesting that COPM are context-dependent. These methods could also be used to investigate university-level students’ rationale towards their change of behaviour, and the influence of socio-demographic characteristics such as age, gender, level of education and socio-economic status on compliance with the COPM. University administrators could then better adapt their public health policies to increase compliance.

### Limitations

One limitation of our study was that because the two observers were randomly assigned different library floors at each time point, only one observer circulated per library floor. Therefore, we could not ensure inter-observer agreement per library floor for any observation time point. Moreover, observers circulated only once per library floor at each time point for the whole observation period. A second limitation of our study concerns students’ gender and age assessment. Because our study was not designed to assess the effect of gender and age, it could not determine if these variables influenced compliance with COPM. A third limitation of our study is that enforcement of COPM was not a parameter measured and analyzed. Consequently, we couldn’t explain with certainty why students were less likely to be compliant with the COPM on Sundays. Perhaps this could be explained by a lower enforcement of COPM that day. A fourth limitation of our study concerns its generalizability to other university libraries and other universities. Nevertheless, our findings provide an encouraging insight into university level students’ compliance with the COPM put in place due to COVID-19, and we believe that they could be complemented with similar observations during other prevention activities implemented in universities.

## Conclusion

University students were highly compliant with COPM in a university library, although there were differences in compliance over time and between weekdays and times of day. These data suggest that in the event of a subsequent SARS-CoV-2 wave, university libraries can remain open, although the latter will need to strengthen COPM at specific time points on specific days, for example, by increasing security surveillance. However, these findings cannot be generalized to other mass-gathering university settings, such as sports facilities and classrooms, as students’ behaviour with COPM might be different. Nevertheless, these findings may support public health authorities in decisions regarding COPMs directed to different universities settings as this method can be applied to focused, rapid observational studies and can lead to data of sufficient statistical power. These findings may also support university administrators in implementing health policies that would lead to the safe resumption of as many in-person activities as possible: a welcome reprieve for university students.

## Annex: Tables

**Table S1:** Non-compliance with proper mask use by week, weekday and time of day, Québec, Canada, March–April 2021

Characteristics	Number (%) of students							
	Non-compliance (N=406, 19.3%)		Compliance (N=1,703, 80.7%)		Non-compliance OR		Non-compliance AOR	
	n	%	n	%	OR	95% CI	AOR	95% CI
Week								
1	104	25.9	297	74.1	1	N/A	1	N/A
2	97	24.1	305	75.9	0.91	0.66–1.25	0.93	0.68–1.29
3	75	13.6	475	86.4	0.45	0.32–0.63	0.47	0.34–0.66
4	130	17.2	626	82.8	0.60	0.44–0.80	0.56	0.42–0.76
Weekday								
Wednesday	106	16.9	521	83.1	1	N/A	1	N/A
Saturday	119	17.9	547	82.1	1.07	0.80–1.43	1.08	0.81–1.45
Sunday	181	22.2	635	77.8	1.40	1.07–1.83	1.47	1.12–1.93
Time of day								
10 a.m.	77	19.9	309	80.1	1	N/A	1	N/A
2 p.m.	173	15.9	913	84.1	0.76	0.57–1.02	0.74	0.55–1.00
6 p.m.	156	24.5	481	75.5	1.30	0.96–1.70	1.22	0.89–1.66

Abbreviations: AOR, adjusted odds ratio; CI, confidence interval, N/A, not applicable; OR, un-adjusted odds ratio

**Table S2:** Total numbers of COVID-19 preventive measures observations by category, Québec, Canada, March–April 2021

Categories	Number of students	% of students
Mask worn correctly and physically distanced	1,653	78.4
Mask worn correctly and not physically distanced	50	2.4
Mask worn incorrectly and physically distanced	392	18.6
Mask worn incorrectly and not physically distanced	14	0.6
Total	2,109	100

Abbreviation: COVID-19, coronavirus disease 2019
